# Acetylation of cell wall is required for structural integrity of the leaf surface and exerts a global impact on plant stress responses

**DOI:** 10.3389/fpls.2015.00550

**Published:** 2015-07-22

**Authors:** Majse Nafisi, Maria Stranne, Lorenzo Fimognari, Susanna Atwell, Helle J. Martens, Pai R. Pedas, Sara F. Hansen, Christiane Nawrath, Henrik V. Scheller, Daniel J. Kliebenstein, Yumiko Sakuragi

**Affiliations:** ^1^Copenhagen Plant Science CenterFrederiksberg, Denmark; ^2^Department of Plant and Environmental Sciences, University of CopenhagenFrederiksberg, Denmark; ^3^Department of Plant Sciences, University of California, DavisDavis, CA, USA; ^4^Department of Plant Molecular Biology, University of LausanneLausanne, Switzerland; ^5^Physical Biosciences Division, Lawrence Berkeley National LaboratoryBerkeley, CA, USA; ^6^Department of Plant and Microbial Biology, University of California, BerkeleyBerkeley, CA, USA; ^7^Danish National Research Foundation Center DynaMOFrederiksberg, Denmark

**Keywords:** cell wall acetylation, trichomes, cuticles, epidermis, *Botrytis cinerea*, peroxidase, mRNA sequencing

## Abstract

The epidermis on leaves protects plants from pathogen invasion and provides a waterproof barrier. It consists of a layer of cells that is surrounded by thick cell walls, which are partially impregnated by highly hydrophobic cuticular components. We show that the Arabidopsis T-DNA insertion mutants of *REDUCED WALL ACETYLATION 2* (*rwa2*), previously identified as having reduced *O*-acetylation of both pectins and hemicelluloses, exhibit pleiotrophic phenotype on the leaf surface. The cuticle layer appeared diffused and was significantly thicker and underneath cell wall layer was interspersed with electron-dense deposits. A large number of trichomes were collapsed and surface permeability of the leaves was enhanced in *rwa2* as compared to the wild type. A massive reprogramming of the transcriptome was observed in *rwa2* as compared to the wild type, including a coordinated up-regulation of genes involved in responses to abiotic stress, particularly detoxification of reactive oxygen species and defense against microbial pathogens (e.g., lipid transfer proteins, peroxidases). In accordance, peroxidase activities were found to be elevated in *rwa2* as compared to the wild type. These results indicate that cell wall acetylation is essential for maintaining the structural integrity of leaf epidermis, and that reduction of cell wall acetylation leads to global stress responses in Arabidopsis.

## Introduction

The epidermis of plants forms a protective layer against xenobiotics, ultraviolet light, and pathogens and provides a waterproof barrier (Liu, 2006; Kourounioti et al., 2013). The epidermis consists of a layer of cells surrounded by thick cell walls that are partially impregnated with the cuticle layer. The main cell wall components in the epidermis are complex polysaccharides: celluloses, hemicelluloses, and pectins. Cellulose forms paracrystalline microfibrils and provides the scaffold of the cell wall; hemicelluloses, mainly xyloglucan in growing tissues such as epidermal cells, crosslink with cellulose to provide support to the cellulose network; while pectins not only crosslink these and other cell wall polymers but also serve as hydrated extracellular matrix components (Carpita and Gibeaut, 1993; Somerville et al., 2004). In contrast, the cuticle consists of highly hydrophobic long-chain hydrocarbons (e.g., cutins and waxes). These cuticle components are transported across the hydrophilic cell wall while partially impregnated within the cell wall (Yeats and Rose, 2013).

Pectins and hemicelluloses are subjected to modifications, of which *O*-acetylation has attracted growing attention in recent years because it significantly impacts a number of industrial applications including food, lumber, and biofuel industries (Klein-Marcuschamer et al., 2010; Gille and Pauly, 2012; Pawar et al., 2013). In pectic homogalacturonan and rhamnogalacturonan (RG) I, *O*-acetylation occurs at the *O*-2 and *O*-3 positions in the backbone galacturonic acid residues (Schols et al., 1990; Ishii, 1997), while in RG II sidechains aceric acid and fucose residues are *O*-acetylated (Whitcombe et al., 1995). In the hemicellulose xyloglucan, acetylation mainly occurs on the galactose residue in the sidechain with the exception that in *Solanaceae* and *Poaceae* glucose residues in the backbones are acetylated (Jia et al., 2005). In the hemicelluloses xylans and gluconomannans *O*-acetylation occurs in the backbone at *O*-2 and *O*-3 position in the xylosyl and mannosyl residues, respectively (Lundqvist et al., 2002; Perrin et al., 2003; Jia et al., 2005; Van Dongen et al., 2011; Gille and Pauly, 2012; Pawar et al., 2013; Xiong et al., 2013). In addition, acetylation of lignins has been reported in some angiosperms (Del Rio et al., 2007; Lu and Ralph, 2008).

Three classes of proteins are known to be involved in *O*-acetylation of cell wall polysaccharides in the Golgi apparatus. The REDUCED WALL ACETYLATION family proteins (RWA1 through 4 in Arabidopsis) are thought to be responsible for the translocation of acetyl-CoA across the Golgi membrane and appear to supply the acetyl-donor to both pectins and hemicellluloses, because knock-out of individual RWAs impacted the level of acetylation in both pectins and hemicelluloses (Lee et al., 2011; Manabe et al., 2011, 2013). For instance, homozygous *rwa2* mutants of Arabidopsis exhibit approximately 20% reduction in the degree of acetylation in both pectins and hemicelluloses (Manabe et al., 2011). The TRICHOME BIREFRINGENCE-LIKE family proteins (TBR1 and TBL1 through 46 in Arabidopsis) are likely to conferr polymer specificity, because different *tbl* mutants of Arabidopsis show polymer-specific reduction in the level of acetylation: *altered xyloglucan 4 (axy4)/tbl27* and *axy4L/tbl22* lack *O*-acetylation of xyloglucan (Gille et al., 2011), while *tbl29/eskimo1(esk1)* mutants have reduced *O*-acetylation of xylan and mannan (Gille et al., 2011; Xiong et al., 2013). Recently, *in vivo* acetyltransferase activity of TBL29 has been demonstrated, lending support to the above notion (Urbanowicz et al., 2014). A gene(s) responsible for pectin-specific acetylation has not yet been identified. Lastly, AXY9, a protein that shows a limited sequence similarity to TBL, was also found to be involved in acetylation of hemicelluloses (Schultink et al., 2015). The reported phenotypes of *rwa2, axy4*, and *tbl29* are diverse; *rwa2* shows enhanced resistance to the necrotrophic fungal pathogen *Botrytis cinerea* (Manabe et al., 2011), *axy4* shows enhanced sensitivity to aluminum (Zhu et al., 2014), while *tbl29* shows enhanced freezing tolerance and dwarfism (Xin and Browse, 1998; Yuan et al., 2013). These observations indicate that cell wall acetylation plays roles in broad aspects of plant stress responses.

The aim of the present work was to gain a better understanding of the role of cell wall acetylation in plant stress responses. To this end, we have carried out a series of phenotypic, microscopic, biochemical and transcriptomic analyses on the panel of Arabidopsis (ecotype Columbia-0) mutants defective in cell wall acetylation. We discovered that in the *rwa2* mutants the architectures of the cell wall-cuticle layer was altered and the trichomes were fragile and collapsed, while the other (*tbl29* and *axy4-3*) mutants appeared similar to the wild type. Furthermore, global transcriptome reprogramming including up-regulation of a large set of stress related genes and the concomitant accumulation of peroxidase activities was observed in the *rwa2* mutant. These effects underpin the importance of cell wall acetylation in the leaf surface integrity and stress responses in plants.

## Materials and methods

### Plant material and growth

*Arabidopsis thaliana* L. Heyn. ecotype Colombia-0 and mutants were grown in soil in Percival climate chamber with a 12 h dark/light cycle at 22°C and 70% relative humidity. When grown on plates, seeds were surface-sterilized as previously described (Weigel and Glazebrook, 2002). Analysis of root growth inhibition was performed according to Weigel and Glazebrook (2002) on half-strength Murashige Skoog (MS) medium containing 0.5% (v/v) sucrose and 0.8% (v/v) agar in the vertically orientation for 1 week. The seedlings were transferred to new MS-agar plates containing hormones (indole-acetic acid and trans-zeatin in the range between 0.01 and 10 μM; ABA in the range between 0.3 and 1.5 μM; JA, 10 and 25 μM), grown vertically. For quantification, the plates were scanned and the root lengths were measured using the software ImageJ (http://rsbweb.nih.gov/ij/).

### Staining and light microscopy

To assess cuticle permeability, detached leaves were floated on the toluidine blue solution, 0.025% (w/v), for 15 min and rinsed with distilled water before imaging with the Stereoscope (Leica EZ4D, Leica, Denmark). Alternatively, soil grown plants were sprayed with the toluidine blue solution, incubated for 45 min and rinsed with excess water to remove the unbound dye.

### Electron microscopy

Leaf pieces of approximately 1 × 3 mm were taken from the tip part leaves having approximately 20 mm long leaf blades from three independent plants in each genotype. Samples were fixed for 4 h in Karnovsky's fixative [5% (v/v) glutaraldehyde, 4% (v/v) paraformaldehyde, 0.1 M sodium cacodylate buffer, pH 7.3], washed in the buffer, and post-fixed in 1% (v/v) osmium tetroxide in the 0.1 M sodium cacodylate buffer for 8 h at 4°C. After washing in the buffer and water, the samples were dehydrated in a graded acetone series and embedded in Spurr resin. The resin was polymerised in an oven at 60°C for 8 h. Ultrathin sections (40 nm thick) were cut with a diamond knife using a Reichert-Jung/LKB Supernova ultramicrotome and sections were contrasted with 1% (v/v) uranyl acetate and lead citrate [2.7% (v/v) in 3.5% (v/v) sodium citrate] and examined in a Philips CM 100 TEM at 80 kV. Quantification of cell wall thickness and cuticle thickness was performed with ImageJ. Five regions from the outer cell wall of the epidermis cells, avoiding trichomes, were analyzed for each genotype, and within each region 20 pairs of measurements were made at the magnification of 64,000 folds. For scanning electron microscopy (SEM), leaf sections were fixed and washed as indicated above, and were dehydrated in ascending concentrations of acetone reaching 100% (v/v) acetone at the final step and dried in an EMS 850 CP drier. Specimens were mounted onto metal stubs, sputter-coated with gold:palladium (1:1) in a Polaron SC 7640 (Quorum Technologies, Newhaven, UK) automated sputter coater, and viewed in a Quanta 200 SEM (FEI CompanyTM) at 10 kV. Contrast adjustments were carried out using Adobe Photoshop CS5 and final mounting of images were done with Adobe Illustrator C52.

### Water loss assay

Rosette leaves from 4-weeks-old plants were cut at the petiole and immediately weighed in plastic weighing boats. The leaves were incubated at room temperature on the laboratory bench and weighed every 20 min. The amount of water loss was calculated as percentage of weight loss compared to original weight.

### Leaf gas exchange

Photosynthesis and transpiration were measured 30 days after germination. A fully expanded leaf, still attached to an intact plant, was placed in the cuvette of a CIRAS-2 portable photosynthesis and transpiration monitor (CIRAS-2 Portable Photosynthesis System; PP Systems, Amesbury, USA). The concentration of CO_2_ in the cuvette was maintained at 400 ppm, humidity at 80% and light level was 150 μmol photons m^−2^ s^−1^. Four independent plants were measured for each line.

### Stomatal aperture measurement

Detached rosette leaves from 4-week old plants were floated in opening buffer (5 mM KCl, 10 mM MES, pH 5.6) for 2 h in the light. Hundred microliters of 50 μM ABA in 2% (v/v) ethanol or 2% (v/v) ethanol (control) was added and the leaves were incubated for 2 h. To measure the response to drought, 4-week-old plants were exposed to 100% humidity for 12 h. Rosette leaves were detached from the plant and incubated on the lab bench for 1 h. Following treatment, the leaves were grinded in opening buffer with a polytron and the homogenate was filtered through nylon cloth (30 μm mesh size). The isolated epidermal fragments were transferred to microscope slides and viewed under the microscope (Leica DM750, Leica, Denmark), 400x magnification.

### Analysis of cuticle composition

To extract wax and cutins, 5–10 leaves per plants were used. The extraction and derivatization of wax and cutin monomers were performed as previously described (Bonaventure et al., 2004; Molina et al., 2006). For the GC-FID analysis the following conditions were used. HP-5 capillary column (30 m, 0.32 mm ID, 0.25 μm film thickness) was used and helium carrier gas at the flow rate of 2 ml min^−1^ with the gradient oven temperature programmed from 140 to 310°C at increment of 3°C min^−1^ followed by 10 min at 310°C. Samples were injected in split mode (30:1 ratio, 310°C injector temperature) and peaks quantified on the basis of their FID ion current. For GC-MS, the same column was used with helium carrier gas at 2 mL min^−1^ and a gradient oven temperature programmed from 110 to 300°C at the increment of 10°C min^−1^. Split-less injection was used and the mass spectrometer operated in scan mode over 40–500 amu (electron impact ionization) with peaks quantified on the basis of their total ion current.

### Pathogen infection assay

Detached leaves of 3–4 weeks old plants were inoculated with *B. cinerea* IK2018 spore solution (5 ^*^ 10^5^ spores ml^−1^ in half strength PDB) as previously described (Denby et al., 2004). To quantify lesion sizes, high quality digital images were acquired and processed with ImageJ.

### mRNA sequencing

Wild type and *rwa2-3* treated with mock (PDB alone) or infected with *B. cinerea* in PDB were planted in a randomized complete block design and individual leaves chosen from different plants. Half of the leaves were infected with *B. cinerea* while the other halves were inoculated with the half strength PDB as a control as previously described (Manabe et al., 2011). Three independent samples per genotype were harvested at 0 h as a control (“untreated”). Thereafter three samples were taken per genotype per treatment at 24 and 48 h post treatment (“mock” and “*B. cinerea*”). Each sample was independently extracted for total RNA using Spectrum RNA kit (Sigma-Aldrich, Denmark) with on-column DNAase treatment and the RNA integrity checked by gel and quantified with NanoDrop 2000 (ThermoScientific, USA). The RNA was then converted into sequencing libraries and sequenced at the Beijing Genome institute. This provides three independent RNAseq samples per genotype per treatment per timepoint. All reads were mapped against a synthetic transcriptome that combined the *A. thaliana* Ensembl (TAIR10) transcriptome with the predicted *Botrytis cinerea* B05.10 transcriptome using TopHat v2.0.8 (Trapnell et al., 2009) and default settings. Mapped reads were counted using HTSeq (Anders et al., 2014) using the setting -m intersection-nonempty. Differential expression was analyzed with EdgeR (Robinson et al., 2010) using a model that accounted for the genotype at *RWA2* and directly tested for an interaction of the genotypes with the treatment (mock vs. *B. cinerea*) and an interaction with time point. All *P* values were adjusted to a FDR of 0.05 within EdgeR using the factorial model and are presented along with the mean corrected cpm per transcript per genotype (Supplementary Table 1).

### Localization of hydrogen peroxide and peroxidase activity

Hydrogen peroxide and peroxidase activity were visualized with the use of 3,3′-diaminobenzidine (DAB; Thordal-Christensen et al., 1997). The DAB precipitate was visualized by light microscopy and stereoscope. Extracellular peroxidase activity was measured using TMB (tetramethylbenzidine) (Barcelo, 1998). Detached rosette leaves from 4-week old plants were either used directly for the assay or incubated with *B. cinerea* or PDB as described above. The leaves were floated on the TMB solution for 30 min. The TMB solution was removed to cuvettes and the absorbance at 654 nm was recorded.

### Expression analysis of the *RWA2* promoter

The DNA fragment covering the 1668 base pairs upstream of the *RWA2* start codon was selected to be the promoter region (Prwa2) and it was PCR amplified using primers with USER overhangs: forward 5′- GGCTTAAUaaattgccttaaatccagcg-3′ and reverse 5′-GGCTTAAUttccgatcagagaagca-3′. The resulting PCR fragment was inserted in the USER cassette of the pLIFE41 vector containing the Kanamycin resistance gene and the BASTA® resistance gene and the resulting vector was introduced in *Agrobacterium tumefaciens*. The wild type Arabidopsis was transformed by floral dipping and BASTA® was used to select for positive transformants and the presence of the Prwa2:GUS construct was verified by PCR with the primers: forward 5′-gttcttattacgacaccg-3′ and reverse 5′-ccggcatagttaaagaaatc-3′.

Histochemical staining was performed as previously described (Harholt et al., 2006).

## Results

### Resistance against *B. cinerea*, surface permeability, and trichomes of acetylation mutants

**Figure 1:**
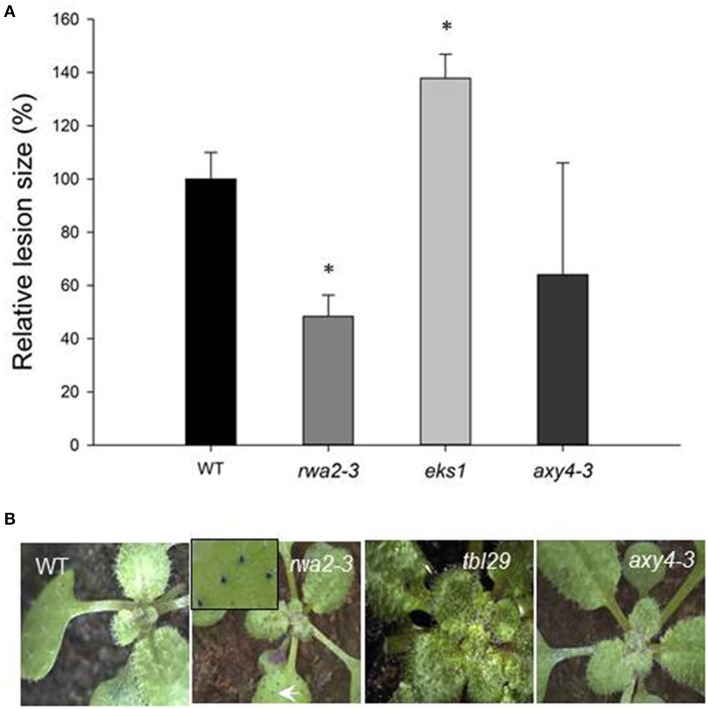
**Resistance to *B*. *cinerea* and toluidine blue staining of wild type and cell wall acetylation mutants (*rwa2-3*, *tbl29*, and *axy4-3)***. **(A)** *B. cinerea* infection defined by lesion area measured. Relative mean values to the wild type are shown and the error bars represent standard deviations (*N* > 8). An asterisk indicates statistically significant difference from the wild type by Student's *t*-test (*P* < 0.05). **(B)** Toluidine blue staining pattern of 3-week old plants. The same results were obtained in three independent analyses. The arrowhead indicates the staining of a trichome.

The responses of the wild type, *rwa2-3, tbl29*, and *axy4-3* mutants to *B. cinerea* were analyzed side-by-side. *rwa2-3* developed smaller lesions as compared to the wild type upon infection (Figure 1A) as previously shown (Manabe et al., 2011). In contrast, *axy4-3* developed lesions comparable in size and appearance to the wild type, whereas *tbl29* developed significantly larger lesions relative to the wild type, indicating that *tbl29* is more susceptible to *B. cinerea* as compared to the wild type.

**Figure 2:**
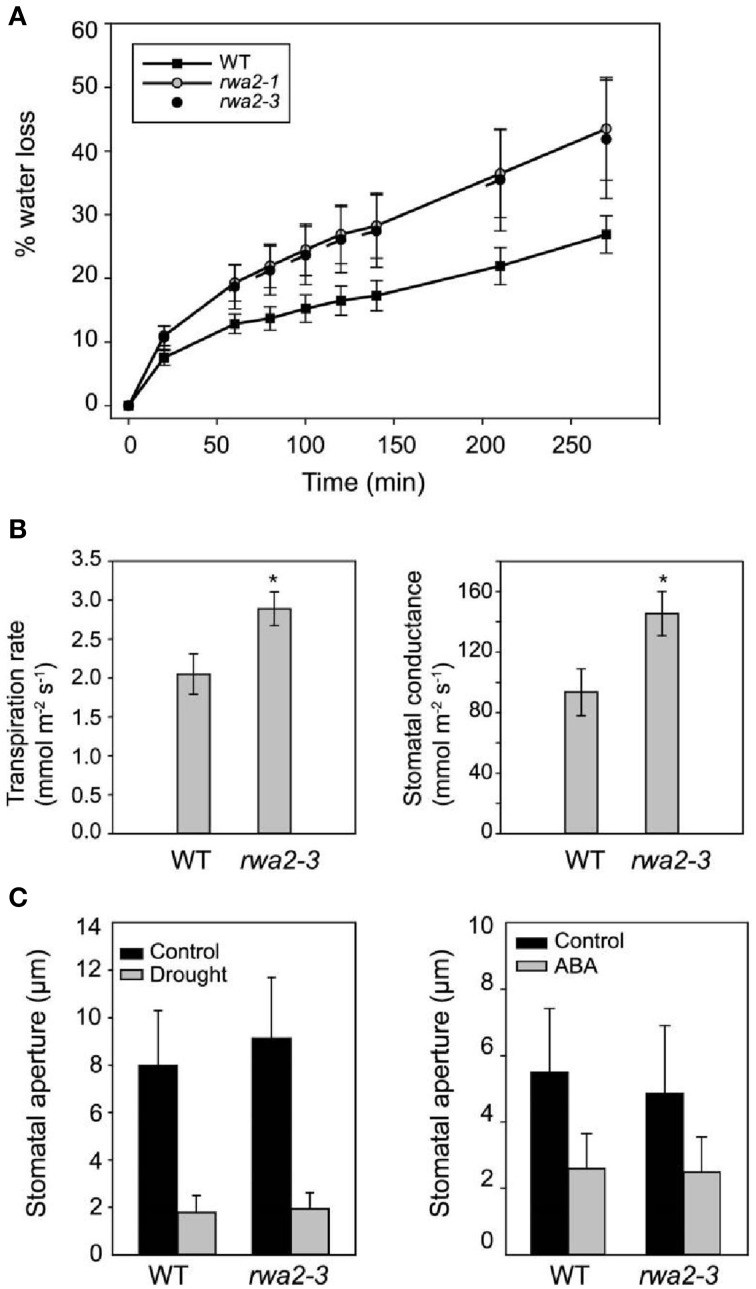
**Enhanced surface permeability in *rwa2***. **(A)** Water loss from detached leaves of wild type, *rwa2-1* and *rwa2-3* plants. Rosette leaves were detached from 4-weeks old plants and incubated on the lab bench. Water loss was determined as percentage weight loss compared to time zero. The data are average ± SD of four leaves per time point. **(B)** Water relations of wild type and *rwa2-3* leaves measured by gas exchange analysis. Values are means ± SD of four leaves from individual plants. **(C)** The aperture of guard cells in response to exogenous ABA and drought. The apertures of guard cells were measured in epidermal peels from leaves treated with 10 μM ABA (left) or in epidermal peels from detached leaves that had been incubated on the bench for 1 h (right) (from plants incubated at high humidity over-night). The data is average of 40 guard cells ± SD. The experiment was repeated three times with similar result. An asterisk indicate a statistically significance difference from the wild type by Student's *t*-test (*P* < 0.05).

Enhanced resistance against *B. cinerea* is often observed among mutants defective in assembly or biosynthesis of the cuticle layer (Chassot et al., 2007; Tang et al., 2007; Voisin et al., 2009; Curvers et al., 2010; Bessire et al., 2011; Suo et al., 2013). In order to test the integrity of the cuticular layers in the acetylation mutants, toluidine blue was applied to the leaf surface. Toluidine blue is a cationic dye that does not stain leaves with an intact cuticular layer due to repulsion by the highly hydrophobic cuticle (Tanaka et al., 2004); however it stains cuticle mutants due to irregularity in the cuticle layer. When treated with toluidine blue, *rwa2-3* retained the dye predominantly in trichomes (Figure 1B). In contrast, neither the wild type, *axy4-3*, nor *tbl29* retained the dye (Figure 1B). In order to further assess leaf permeability of the *rwa2* mutants, detached leaves of *rwa2-1* and *rwa2-3* were incubated at room temperature and the weight loss was measured over time. The *rwa2* mutants lost weight faster than wild type (Figure 2A). Leaf gas exchange measurements were conducted in order to quantify transpiration rate and stomatal conductance across lamina. Transpiration rate is a measure of the actual net water loss, while stomatal conductance is a measure of conductivity for water transport across lamina, and both measures depends on the ratio of open/closed stomata but also on the integrity of the cuticle layer. Since the phenotypes of *rwa2-1* and *rwa2-3* were largely indistinguishable, only *rwa2-3* was analyzed. The stomatal conductance and transpiration rate were increased up to 50% of the wild-type level in *rwa2-3* (Figures 2B,C). It should be noted that the increased water loss and transpiration is not due to misregulation of guard cells as no difference in the stomatal aperture was observed between the wild type and *rwa2* neither under drought conditions nor upon treatment with abscisic acid (ABA), which is a major regulator of stomata closure (Acharya and Assmann, 2009) (Figure 2C). There was no difference in the stomatal density between the wild type and *rwa2-3* (data not shown). These results show that the leaf surface of *rwa2*, particularly in trichomes, is damaged and more permeable to transpiration.

**Figure 3:**
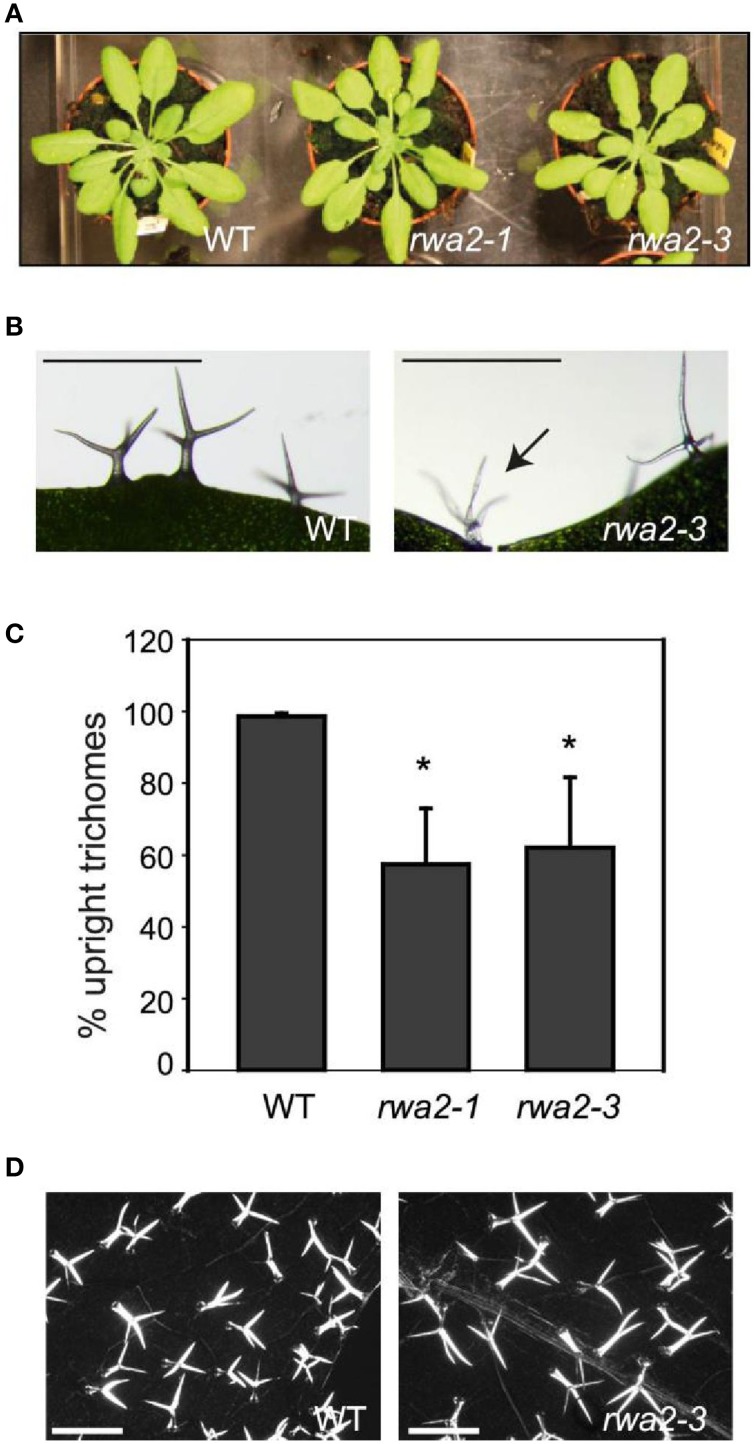
**Trichome integrity is altered in *rwa2***. **(A)** Macroscopic appearance of 3-weeks old wild type, *rwa2-1* and *rwa2-3* grown in growth chamber with 12 h dark/light cycle. **(B)** Trichomes of the wild type (right) and *rwa2-3* (left). The arrow indicates a collapsed trichome on *rwa2-3*. The scale bar equals 0.5 mm. **(C)** The percentage of “normal looking” trichomes [as depicted in (**B** left)] on the leaves of wild type, *rwa2-1* and *rwa2-3*. Leaves were destained in ethanol followed by quantification of the total number of trichomes and the number of collapsed trichomes when viewed under the microscope. The data is the average of three independent experiments ± SD. The number of non-collapsed trichomes on *rwa2-1* and *rwa2-3* leaves are significant different from wild type as tested by Student's *t*-test (*P* < 0.05) as indicated by the asterisks. **(D)** Trichome birefringence of the wild type (left) and *rwa2-3* (right) leaves. Scale bar equals 1 mm.

Impairment in cuticle integrity is also known to cause collapsed trichomes. Many trichomes on *rwa2* leaves were found to be thinner and more transparent as compared to the wild type and were often partially or fully collapsed (Figures 3B–D). The number of fully collapsed trichomes was about 30% of the total number of leaf trichomes in *rwa2* while no collapsed trichomes were observed on wild-type leaves (Figure 3C). The remaining 70% of the *rwa2* trichomes were not collapsed but often appeared fragile. Macroscopically the *rwa2* mutant plants (*rwa2-1* and *rwa2-3*) appear comparable to the wild-type plants under the standard growth conditions in the green house and growth chambers (Figure 3A). Collapsed trichomes has also been seen in the *tbr*-2 mutant (Suo et al., 2013), wherein the deposition of paracrystalline cellulose is impaired in trichomes, resulting in loss of trichome birefringence under UV light (Potikha and Delmer, 1995). Trichome birefringence of *rwa2* appeared indistinguishable from that of wild type under the conditions tested (Figure 3D). Apart from these morphological differences, trichomes on *rwa2* leaves develop normally and have the typical three-branched structure with a similar height to those in the wild type.

### Cuticle and cell wall architectures and trichome morphology are altered in *rwa2*

**Figure 4:**
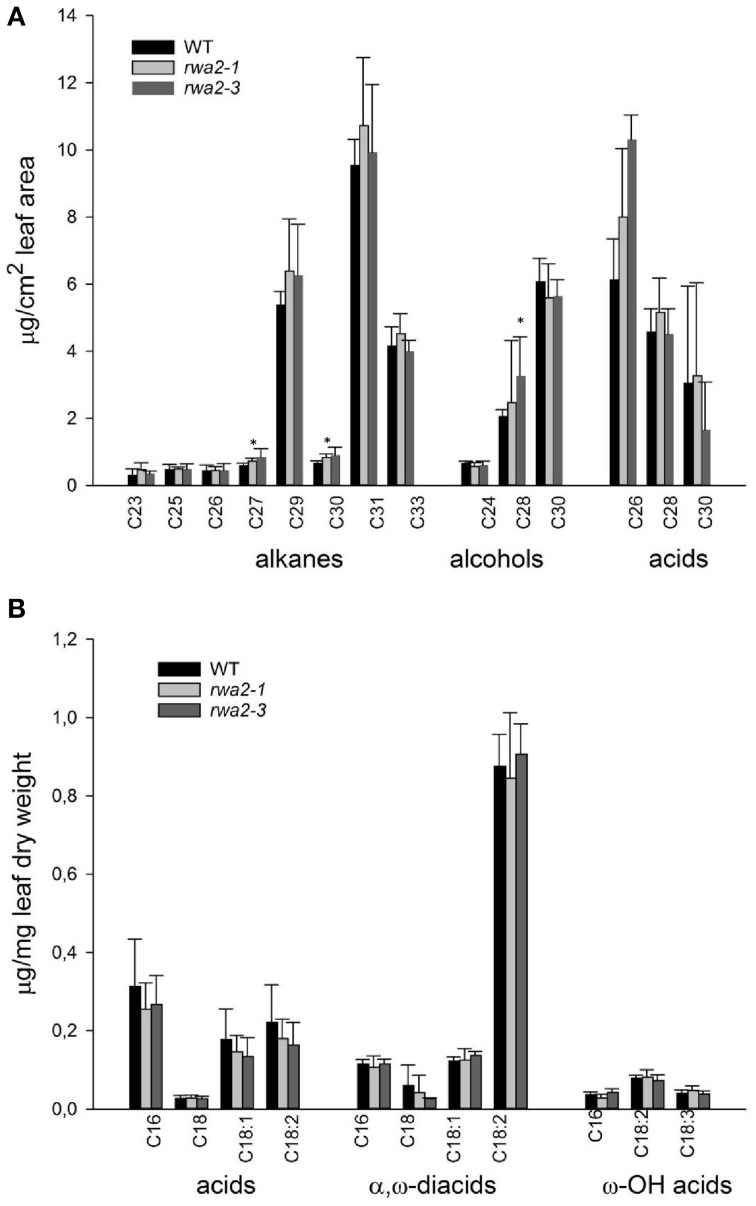
**Epicuticular wax and cutin composition analysis of leaves of 4-weeks old wild type, *rwa2-1* and *rwa2-3* plants. (A)** Composition of epicuticular waxes extracted by dipping in chloroform and derivatized with BSTFA. **(B)** Composition of depolymerized residual-bound lipids extracted extensively with methanol/chloroform followed by NaOMe-catalyzed acetylation. Values are means of at least five independent samples and the error bars represent the standard deviations. An asterisk indicate a statistically significance difference from the wild type by Student's *t*-test (*P* < 0.05).

To test if cuticle content and/or composition have been affected in *rwa2*, the chemical composition of the cuticle was determined by gas chromatography (Bessire et al., 2007). No significant difference in the composition or content of the cuticular wax and cutin monomers was observed between the wild type and the *rwa2* mutants (Figures 4A,B). Therefore, biosynthesis and delivery of the wax and cutin components appear to occur normally in *rwa2*.

**Figure 5:**
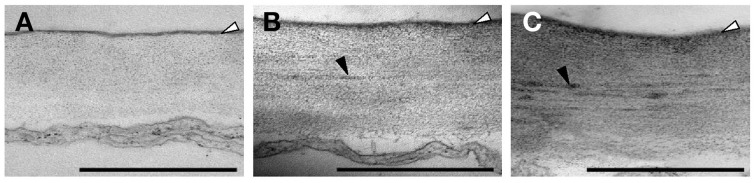
**Ultrastructural organization of the epidermis**. Electron microscopic images of ultra-thin sections of the outer cell wall in the upper leaf epidermis were obtained for the wild type **(A)**, *rwa2-1* **(B)**, and *rwa2-3* **(C)**. The cuticle layer is indicated by the white arrowheads. The electron dense deposits are indicated by black arrowheads. All scale bars equal 500 nm.

In order to gain insights into the ultrastructure of the cuticle and cell wall, transmission electron microscopy (TEM) was performed on the leaf epidermal cells. In the wild type, the cuticle appeared compact and highly electron dense while the cell wall layer underneath was relatively electron opaque (Figure 5A). The cuticle layers in *rwa2-1 and rwa2-3* appeared notably more diffused than that in the wild type (Figures 5B,C) with the average thickness increased by approximately 50% of that of the wild type (Table 1). The similar thickness values and the difference between the genotypes were observed in two independent experiments using independently grown plants. Apart from the change in the cuticle layer, the cell wall layer in *rwa2* showed marked difference from the wild type. The cell wall layer in *rwa2* was interspersed with electron-dense deposits, which were rarely seen in the wild type (Table 1). In addition, the average cell wall thickness in *rwa2* was approximately 30–50% larger than that in the wild type (Table 1). Hence, we conclude that both the cuticle and cell wall ultrastructures were altered in *rwa2*.

**Table 1:** **Ultrastructure of the cell wall and cuticle layer in the wild type and the *rwa2* mutants**.

**Strain**	**^a^Thickness (nm)**		**^b^Number of electron dense deposits per image**	**^c^Thickness of the trichome base (nm)**
	**Cell wall**	**Cuticle layer**		
Wild type	417 ± 93	19 ± 5.0	0.83 ± 1.2	67 ± 0.67
*rwa2-1*	558 ± 109^*^	30 ± 8.0^*^	7.3 ± 4.2^*^	ND
*rwa2-3*	642 ± 154^*^	32 ± 8.0^*^	11 ± 8.6^*^	100 ± 13^*^

a *Three hundred randomly selected positions derived from three independent plants were analyzed*.

b *Six independent, equal sized images obtained at the same magnification were analyzed*.

c *Three independent sections, each cutting through the middle of a trichome, were analyzed*.

* *Student's t-test, P < 0.05. ND, not determined*.

**Figure 6:**
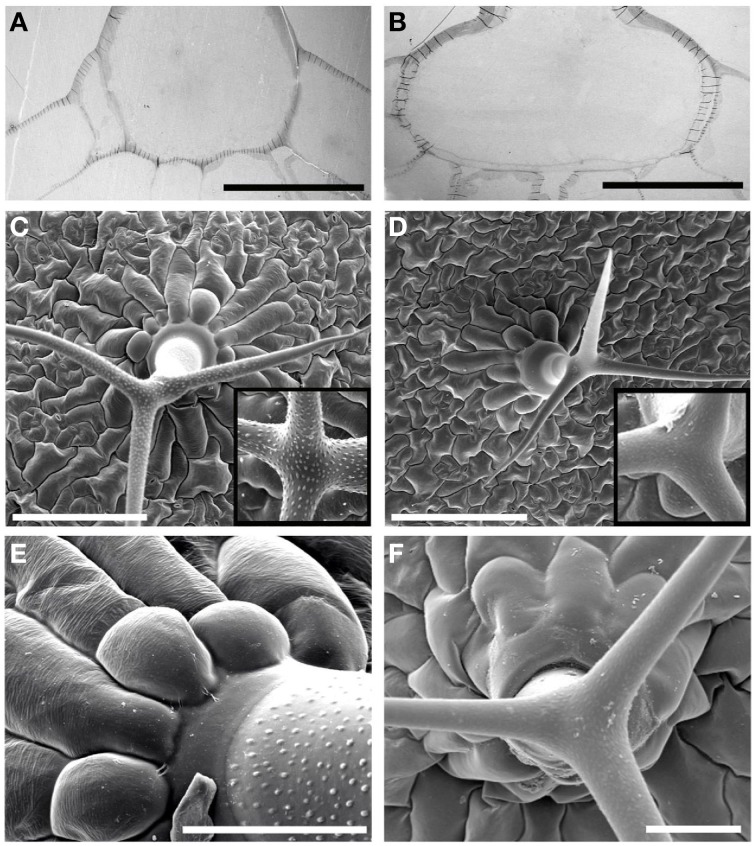
**Ultrastructural organization of the trichomes**. Transverse ultra-thin sections of the trichomes in the wild type **(A)**, and *rwa2-3* **(B)**. Scale bars equal 50 μm. Lines across the cell wall are artifacts due to wrinkling of the ultra-thin sections. Scanning electron micrographic images of trichomes in the wild type **(C)** and the *rwa2-3* **(D)**. Scale bars equal 200 μm. Inserts (100 × 100 μm) represent close-up images of trichome branches. Close-up images of the trichome base in the wild type **(E)** and *rwa2-3* **(F)**. Scale bars equal 50 μm. All images were acquired by electron microscopy.

Given the enhanced permeability and structural impairments of *rwa2* trichomes (Figures 1B, 3B), the traverse sections of trichomes were analyzed by TEM. It was noticed that the base of the trichomes in *rwa2-3* was notably larger than that in the wild type (Figures 6A,B). The average width of three traverse sections, each cutting through the middle of a trichome increased in *rwa2-3* by 50% of the wild type level (Table 1). The increase in size of the *rwa2* trichome base was confirmed by SEM of trichomes (Figures 6C,D). SEM analysis revealed additional anatomical differences between the wild-type and *rwa2-3;* the papillae were either entirely missing or reduced in size in *rwa2-3* as compared to the wild type (Figures 6C–F). Furthermore, in some cases the trichome base in *rwa2-3* was sunken (Figure 6F).

### Global transcriptomic reprogramming occurred in *rwa2*

**Figure 7:**
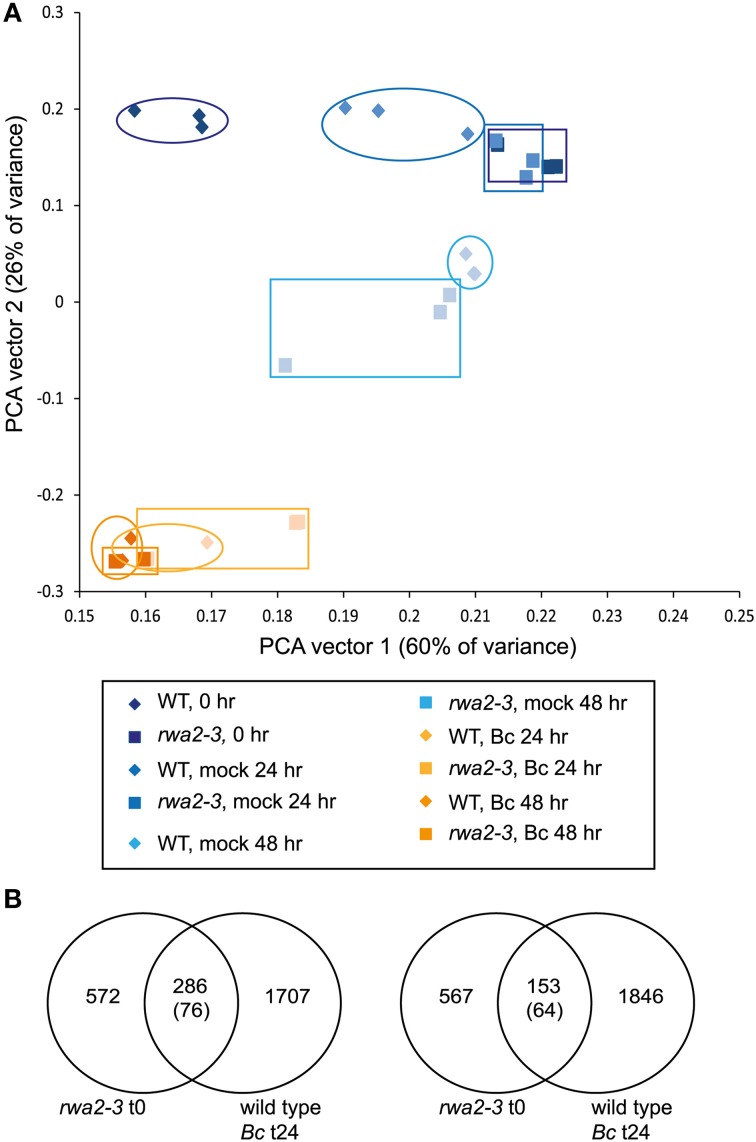
**Global transcript analysis. (A)** Shown is a principal component analysis of the entire dataset with the individual samples plotted. Wild type samples are shown as diamonds and *rwa2* samples are shown as squares with the blue to orange transition showing the time course of the experiment. The first two PCA vectors are utilized that explain 86% of the total variance. **(B)** VENN diagrams showing the overlap of genes differentially expressed (left side: upregulated genes, right side repressed genes) in *rwa2* control vs. wild type control, and wild type infected with *B. cinerea* vs. wild type mock across the time points. The genes that are differentially expressed in *rwa2* control vs. the corresponding wild-type samples have a high overlap with the expression profile of genes in wild type infected with *B. cinerea*. The numbers in brackets represent the overlap expected by chance. The overlaps are highly significant both for induced (*P* < 0.001) and for repressed genes (*P* < 0.001) as determined by χ^2^-test.

We hypothesized that the surface damage caused by the *rwa2* mutation could have a significant impact on plant stress responses and that transcriptome profiling would shed light on which stress response(s) is affected. Wild-type and *rwa2-3* leaves were either untreated or treated with mock [potato dextrose broth (PDB) only] or *B. cinerea* spore solution in PDB for 24 and 48 h and mRNA sequencing was performed (Supplementary Table 1). The largest difference in the transcriptome profile was observed between the untreated wild type and *rwa2-3*, indicating that the untreated *rwa2-3* perceives the environment differently from the wild type (Table 2). Out of 21,178 transcripts that were sequenced in both the wild-type and *rwa2*-3, 1650 transcripts showed statistically significant differential abundance: 857 genes up-regulated and 763 genes down-regulated [log_2_ fold ≥ 2, false discovery rate (FDR) ≤ 0.05]. The mock and *B. cinerea* treatments led to smaller differences between *rwa2-3* and the wild type in terms of the number of genes with altered expression levels at both 24 h and 48 h after infection (Table 2). Principal component analysis showed that genotype and treatments describe 86% of the total transcriptomic variance detected (Figure 7). The first vector (PCA vector 1) largely describes the differences between the genotypes at the untreated and early mock treated samples (untreated wild type vs. *rwa2-3*), while the second vector (PCA vector 2) largely describes the response of the genotypes to infection with *B. cinerea* (untreated, mock, and *B. cinerea* treatments). In the wild type, the mock treatment (detachment of leaves followed by incubation in a water-agar medium and application of PDB) caused a notable change in the transcriptome profiles. Interestingly, both the untreated and mock-treated *rwa2-3* for 24 h showed a significant overlap with the mock-treated wild type. Upon treatment with *B. cinerea*, the transcriptomes of the wild type and *rwa2-3* showed an even higher degree of overlap (Figure 7). This indicates that the *rwa2-3* responds to *B. cinerea* similarly to the wild type even though their initial transcriptomes are highly divergent.

**Table 2:** **Number of genes with altered expression in *rwa2* as compared to the wild type as identified by mRNA sequencing (log_2_ fold ≥ 2, FDR ≤ 0.05)**.

**Treatment**		**Up-regulated**	**Down-regulated**
Untreated		857	793
Mock treatment	24 h	320	40
	48 h	134	10
Botrytis treatment	24 h	415	319
	48 h	32	5

Because the major difference was found in the untreated *rwa2-3* and wild type, further analysis focused on these samples. Analysis of gene ontology (GO) categories by the AmiGo software (Ashburner et al., 2000) showed that a large fraction of the up-regulated genes in *rwa2-3* belong to categories relating to both abiotic and biotic stress responses, with notable examples of responses to and transport of organic and inorganic substances, as well as detoxification processes and oxidative stress responses (Supplementary Table 2). Several of the genes that are up-regulated in untreated *rwa2-3* have been shown to be important for resistance against *B. cinerea* (Table 3 and references therein). This includes lipid transfer proteins (AT4g12470, AT4G12480, AT4g12490) and peroxidases (AT2g37130, AT5g39580, and AT5g64129) that were up-regulated in a cutinase-overexpressing Arabidopsis transgenic line as compared to the wild type (Chassot et al., 2007). Moreover, overexpression of each of these transcripts in the wild-type background was sufficient to cause enhanced resistance against *B. cinerea* (Chassot et al., 2007). Hence the enhanced resistance of *rwa2-3* against *B. cinerea* can be explained by the constitutive and coordinated up-regulation of these defense related genes.

**Table 3:** **Defense related genes upregulated in the untreated *rwa2-3* as compared to the untreated wild type (*N* = 3, FDR < 0.05)**.

**Gene description**	**Locus tag**	**Fold change rel. to WT**
Lipid transfer proteins	At4g12480^a^,^b^	301
	At4g12490^a^	492
	At2g38530^a^	10
	At4g12470/AZI1^a^	37
	At2g37870^c^	111
Peroxidases	At2g37130^a^	5.5
	At5g64120^a^	6.5
Defensins	At5g44430/PDF1.2C^d^	4.5
	At1g75830/PDF1.1^d^	315
Protease inhibitors	At2g38870^a^	5
	At5g43580^e^	256
Trypsin inhibitors	At2g43510^a^	10
	At1g73260^f^	12
ELI-3 defensive protein	At4g37990^g^	10.5
PR4	At3g04720^h^	3
GDSL lipase 1 (GLIP1)	At5g40990^i^	96
PROPEP3	At5g64905^j^	12
Glycolate oxidase 3 (GOX3)	At4g18360^k^	41

a *Chassot et al., 2007*,

b *Li et al., 2012*,

c *Hernandez-Blanco et al., 2007*,

d *De Coninck et al., 2010*,

e *Laluk and Mengiste, 2011*,

f *Li et al., 2008*,

g *Kiedrowski et al., 1992*,

h *Thomma et al., 1998*,

i *Oh et al., 2005*,

j *Huffaker et al., 2006*,

k *Rojas et al., 2012*.

In contrast to the up-regulated GO categories, the most overrepresented biological process category among the down-regulated genes was “response to hormones” (Supplementary Table 3). Manual inspection of genes that specifically respond to treatment with auxin, ABA, brassinosteroid (BR), cytokinin (CK), ethylene, gibberellic acid, and jasmonic acid (JA) (Nemhauser et al., 2006) showed that several transcripts that are up-regulated upon treatment with CK, auxin, and JA were coordinately down-regulated in *rwa2-3* untreated leaves (Supplementary Figure 1). However, the *rwa2-3* mutant was found to retain the wild-type level of response to these hormones; when seedlings were grown on nutrient agar plates supplemented with the hormones JA, CK, auxin or ABA no difference in general growth or root inhibition was observed between wild type and *rwa2-3* (Supplementary Figure 2).

The transcript profile of the untreated *rwa2-3* relative to the untreated wild type was compared to publically available microarray data by using Signature Tool in the Genevestigator software (Zimmermann et al., 2004). The 300 genes that showed the highest differential expression as compared to the wild type and with the lowest FDR values, hence highest confidence, were subjected to the analysis. Among the top 30 transcript datasets that showed similarities to the *rwa2-3* transcript profile, experiments inducing oxidative stress (growth under high light, cold, drought; CAT2HP1 overexpressor; *catalase2-1*) were highly represented (Supplementary Table 4). In addition, consistent with the GO analysis, transcript profiles upon changes in lipid metabolism or signaling (*suppressor of SA insensitivity-1*, application of phytoprostane A1), exogenous application of xenobiotics (phenanthrene, fenclorin, sulfometuron methyl), alteration of phytohormones (ARR22 overexpression, application of salicylic acid), and microbial infection (*Golovinomyces cichoracearum, Alternaria brassicicola*) were found.

### Extracellular peroxidases accumulate in the *rwa2* mutant

**Figure 8:**
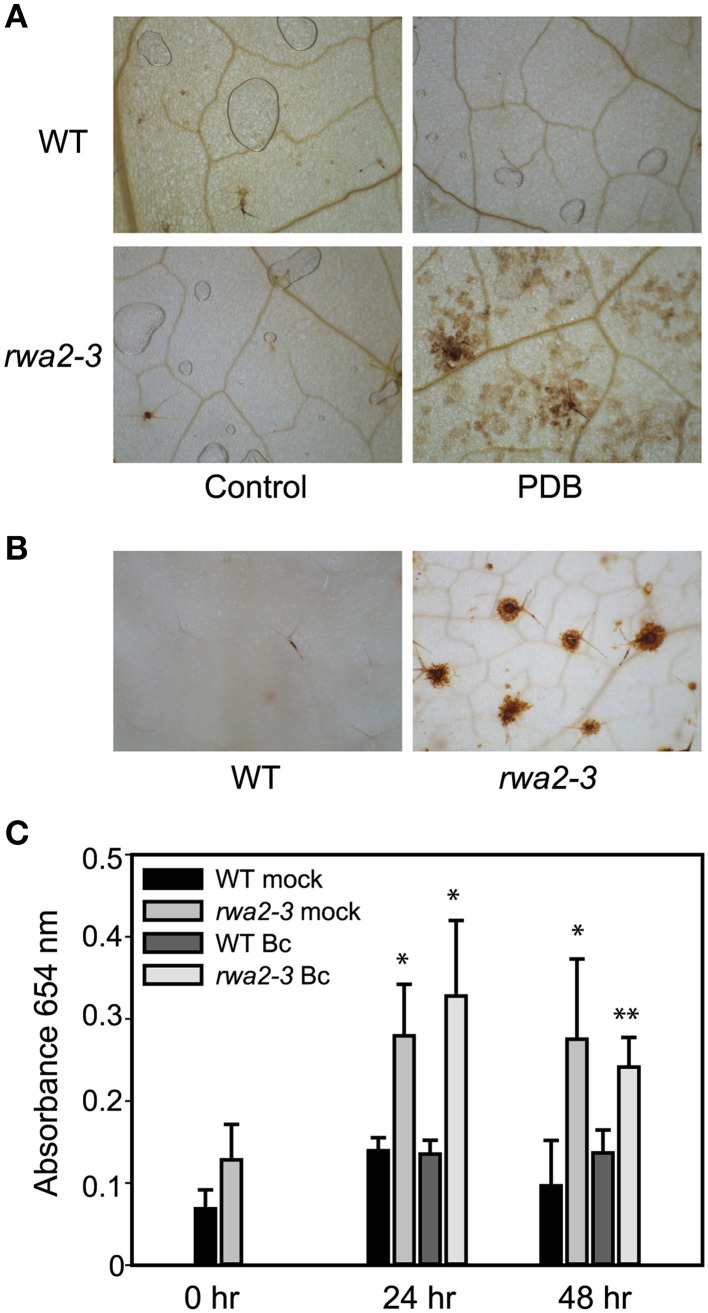
**Increased peroxidase activity and hydrogen peroxide accumulation in *rwa2*. (A)** DAB staining for hydrogen peroxide in untreated (left) and in response to treatment with potato dextrose broth (PDB) (right). Five micro liters of 2% (v/v) PDB was placed on each side of the mid vain for 48 h followed by DAB staining. Chlorophylls were extracted in 96% (v/v) ethanol. **(B)** Peroxidase activities accumulate at trichome bases in uninfected *rwa2* leaves. Leaves were stained with DAB in the presence of 0.1% (v/v) H_2_O_2_ for 1 h. Chlorophylls were extracted in 96% (v/v) ethanol. **(C)** Extracellular peroxidase activity in the wild type and *rwa2-3* leaves. Detached leaves were treated with mock (PDB) or *B. cinerea* for 24 and 48 h. To measure peroxidase activity, the leaves were floated with the adaxial side downwards in TMB and H_2_O_2_. Peroxidase activity was measured as absorbance at 654 nm. The data is average of three samples ± SD. The asterisks indicate significant difference between wild type and *rwa2-3* as determined by Two-Way ANOVA test (^*^P < 0.05; ^**^P < 0.01).

To test if *rwa2* experiences increased oxidative stress, we conducted 3,3′-diaminobenzidine (DAB) staining to visualize H_2_O_2_ production (Thordal-Christensen et al., 1997). DAB staining did not show notable accumulation of H_2_O_2_ on the leaf surface of the wild type and the *rwa2* mutants (Figure 8A). On the other hand, the mock treatment (PDB) resulted in an elevated H_2_O_2_ production in *rwa2-3* leaves as compared to the wild type (Figure 8A). To test peroxidase activities, the leaves from the same developmental stage were incubated with DAB in the presence of H_2_O_2_ as previously described (Thordal-Christensen et al., 1997). Intense DAB staining around the trichome base was observed for untreated *rwa2-3* leaves whereas no notable staining was observed for the wild type (Figure 8B). Consistently, the measurement of extracellular peroxidase activity with the tetramethylbenzidine assay (Barcelo, 1998) shows that the *rwa2-3* mutant possesses a slightly higher extracellular peroxidase activity as compared to the wild type (Figure 8C, 0 h), and the activity increased further by 24 h upon treatment with mock (PDB alone) or *B. cinerea* spores in PDB to the comparable levels. It should be noted that the leaves at the same developmental stage were used and there is no difference in leaf sizes between the wild type (1.77 ± 0.23 cm^2^, *N* = 10) and the *rwa2-3* mutant (1.92 ± 0.33 cm^2^, *N* = 11). Therefore, the *rwa2* mutant accumulates a higher level of peroxidase activity around the trichome base and the activity is further induced upon treatment by components in PDB.

### The promoter of *RWA2* is active in trichomes

**Figure 9:**
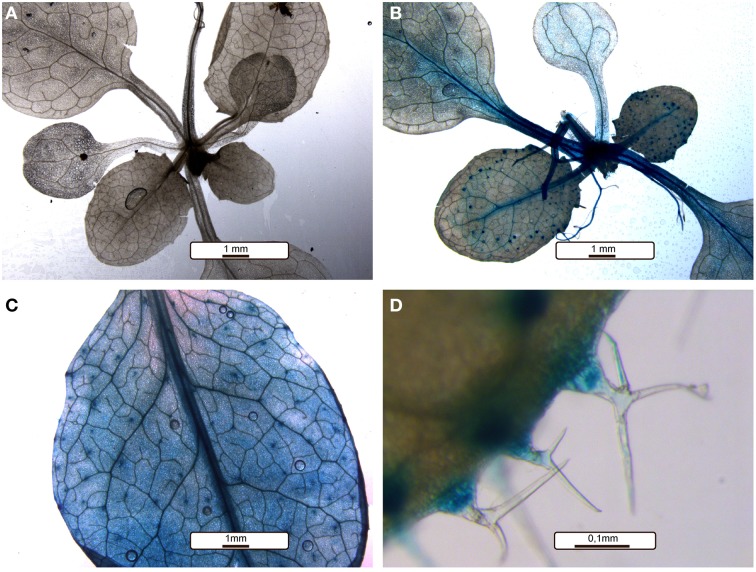
***RWA2* is expressed abundantly in leaves, particularly in trichomes in Arabidopsis**. *RWA2* expression was analyzed by GUS staining in 3-weeks old Arabidopsis plants, 10 independent transformed lines were tested. **(A)** The negative control (promoter-less GUS line). **(B)** A representative *RWA2* promoter*:GUS* fusion line. **(C,D)** Close-up images of leaves and trichomes in a representative *RWA2* promoter*:GUS* fusion line.

*RWA2* is expressed in root, stem, leaf and flower (Lee et al., 2011; Manabe et al., 2011) with specific expression in the xylem in roots and secondary xylem in stem (Lee and Rose, 2011; Manabe et al., 2011). However, tissue-type specific expression pattern of *RWA2* in leaves has not yet been reported. Ten independent lines of transgenic lines expressing a transcriptional fusion of the *RWA2* promoter region (from −1600 to +50 bp relative to A in the start codon) and the reporter gene β-glucuronidase (GUS) were analyzed. As shown in Figure 9, histochemical staining with 5-Bromo-4-chloro-3-indolyl-β-d-glucuronide revealed promoter *RWA2:GUS* expression in leaves and notably in trichomes. In light of the *rwa2* trichome phenotypes, strong GUS expression in this tissue supports the notion that *RWA2* expression is required for structural integrity of leaf surface and particularly trichome bases.

## Discussion

We discovered that the structural integrity of Arabidopsis leaf surface was severely impaired in *rwa2* mutants. The cell wall layer in the epidermis was morphologically distinct from that of the wild type with accumulation of numerous electron-dense deposits (Figure 5), indicative of abnormal cell wall architecture. Notably, *rwa2* showed enhanced permeability to the cationic dye toluidine blue exclusively in trichomes (Figure 1), and trichomes appeared fragile; the base of the trichome was enlarged, often either ruptured or sunken (Figure 6) and a large fraction of the trichomes on *rwa2* was collapsed (Figure 3). In addition, the papillae on the trichome surface were dramatically reduced in size (Figure 6). These phenotypes are often associated with mutants that are defective in cuticle integrity and biosynthesis (i.e., transgenic CUTE plants that overexpresses a cutinase and *lacs2, lcr, fdh, pec1*, and *gl1* mutants) (Serrano et al., 2014). Although the cutin monomer and wax compositions were not altered in *rwa2* as compared to the wild type, the cuticle layer in epidermis was notably diffused and thicker (Table 1, Figure 5). Moreover, mRNA sequencing revealed a massive reprogramming of the transcriptome in the *rwa2-3* mutant characterized by coordinated induction of genes related to abiotic and biotic stress responses (Table 3, Figure 7). These results demonstrate that cell wall acetylation plays an important role in maintaining the surface structural integrity in Arabidopsis leaves and its impairment results in global stress responses in plants. Because it has previously been proposed that RWA2 functions as an acetyl-CoA transporter localizing to the Golgi apparatus (Manabe et al., 2011), it was possible that the cytosolic acetyl-CoA pool had been altered in *rwa2* and this might have caused the observed phenotypes. In the cytosol, acetyl-CoA is converted to malonyl-CoA that feeds into the synthesis of a range of phytochemicals including wax and cutins (Oliver et al., 2009). It has been shown that overexpression of an ATP citrate lysase that synthesizes acetyl-CoA in the cytosol of Arabidopsis resulted in 30% increase in wax loading and the cutin monomer octadecadie-1,18-dioic acid content (Xing et al., 2014). In contrast, *rwa2* contained the wild-type level of cutin and wax components (Figure 4). Therefore, it was concluded that the cytosolic acetyl-CoA pool is unlikely to be altered at a significant extent and to cause the observed phenotypes.

Because the increased permeability and enhanced resistance to *B. cinerea* were only observed in *rwa2* and not in *tbl29* and *axy4-3* (Figure 1), we speculate that the reduced acetyl groups in pectins contribute to the observed phenotypes by interfering with the normal cell wall and cuticle assembly. Earlier studies have shown that during the development of the cuticle, cutin polymers become progressively impregnated with the cell wall polysaccharides, particularly pectins (Schieferstein and Loomis, 1959). Pectinase treatment has been reported effective for releasing the cuticles (Orgell, 1955; Baker and Procopiou, 1975) and isolated cuticle from pear leaf stains with ruthenium red, a stain widely used to detect pectins (Norris and Bukovac, 1968). Treatment with EDTA and oxalic acid or ammonium oxalate, which are often used for isolation of pectins, have also been shown to be effective in isolating cuticles (Huelin and Gallop, 1951). In *rwa2*, the excess hydroxyl groups, as a result of reduced acetylation, may form atypical crosslinking with the cutins leading to abnormal cuticle assembly. Alternatively, though mutually nonexclusive, cell wall acetylation may impact the transport of cutin and wax across the cell wall. Both cutins and waxes are long chain fatty acid derivatives and must cross the highly hydrophilic cell wall layer and the transport process is thought to involve apoplastic carriers, possibly lipid transport proteins (LTPs) (Yeats and Rose, 2013; Hurlock et al., 2014). Notably, the expression levels of 10 genes coding for LTPs and LTP like proteins were shown to be up-regulated in *rwa2* as compared to the wild type, which may represent a compensatory response (Table 3).

Transcriptomic profile of the *rwa2*-3 mutant was dramatically different from that of the wild type (Table 2, Figure 7) with a coordinate up-regulation genes involved in responses to detoxification and oxidative stress (Supplementary Table 2). Trichomes are involved in a range of protective mechanisms from adverse environmental conditions including protection from UV and excessive light (Karabourniotis and Bornman, 1999; Franke et al., 2005), and heavy metals detoxification (Freeman et al., 2006; Sarret et al., 2009; Marmiroli et al., 2010). Moreover, disturbance of cuticle biosynthesis or overexpression of a cutinase, as well as mechanical stress of leaf epidermis (i.e., wounding by forceps, soft rubbing by fingers), have been shown to induce reactive oxygen species and resistance to *B. cinerea* (L'Haridon et al., 2011; Benikhlef et al., 2013). It is likely that altered cell wall and cuticle assembly, as discussed above, causes structural and functional impairments in the trichomes and cuticle layer causing the induction of detoxification and oxidative stress responses, and this may ultimately lead to enhanced resistance against *B. cinerea* in *rwa2*. This notion is further supported by the previous results that ectopic overexpression of individual peroxidase genes induced in *rwa2-3* was sufficient to cause enhanced resistance against *B. cinerea* in Arabidopsis (Chassot et al., 2007). Peroxidases are thought to cause cell wall stiffening in the presence of H_2_O_2_ through oxidative formation of covalent bonds between aromatic cell wall components (Francoz et al., 2015 and reference therein). It is possible that the induced levels of H_2_O_2_ and peroxidases (both transcripts and activities) (Figure 8, Table 3) might cause an enhanced cell wall fortification in and around the trichomes in the *rwa2* mutants, causing restricted infection by the fungus.

## Author contributions

All authors contributed to either the conception, design of the work or the acquisition, analysis and interpretation of data, drafted and approved the manuscript.

### Conflict of interest statement

The authors declare that the research was conducted in the absence of any commercial or financial relationships that could be construed as a potential conflict of interest.
