# Supplementary material for: Acetylation of cell wall is required for structural integrity of the leaf surface and exerts a global impact on plant stress responses
Source: Front Plant Sci. 2015 Jul 22;6:550. doi: 10.3389/fpls.2015.00550 (PMC4510344; doi:10.3389/fpls.2015.00550)
Supplement: Supplementary file 4 [file Table4.DOCX]

| **Supplementary Table 4: Signature profile of the transcript profile of the untreated *rwa2*-3 relative to the untreated wild type.** |  |
| --- | --- |
|  |  |
|  |  |
| **Experiment** | **Similarity** |
|  |  |
| ARR22 OEX/Col-0 | 1.229 |
| CAT2HP1/col-4 | 1.202 |
| salicylic acid/mock treated seedlings | 1.190 |
| fenclorim (4h)/solvent treated root culture samples | 1.182 |
| CAT2HP1/col-4 | 1.180 |
| des1-1/col | 1.170 |
| G. cichoracearum (96h)/non-infected whole rosette leaves | 1.166 |
| fenclorim (24 h)/solvent treated root culture samples | 1.163 |
| K16331/BIG | 1.152 |
| shift high CO2/SD to air CO2/SD (cat2-1)/high CO2/SD (cat2-1) | 1.149 |
| callus formation (12h)/untreated root samples | 1.148 |
| sulfometuron methyl (24h)/mock treated leaf samples | 1.147 |
| high light study 2 (3h)/untreated leaf samples (col-4) | 1.141 |
| light/drought (aox1a/untreated leaf samples (aox1a) | 1.140 |
| CAT2HP1/col-4 | 1.139 |
| high light study 2 (3h)/untreated leaf samples (CAT2HP1) | 1.137 |
| shift high CO2/SD to air CO2/SD (cat2-1)/high CO2/SD (cat2-1) | 1.136 |
| CMP (4h)/solvent treated root culture samples | 1.136 |
| ssi2-1/col | 1.133 |
| cat2-1/col | 1.131 |
| (NH4)2SO4/NH4NO3 (col) | 1.131 |
| A. brassicicola study 3 (col)/mock treated samples | 1.129 |
| high light study 3 (8 h)/untreated leaf samples | 1.129 |
| phytoprostane A1 (cell culture)/solvent treated cell culture samples | 1.123 |
| light/drought (aox1a/untreated leaf samples (aox1a) | 1.122 |
| light/low CO2/dark/low CO2 | 1.121 |
| phenanthrene/untreated col plant samples | 1.121 |
| pvip1:pvip2/col | 1.120 |
| P. syringae pv. Tomato (DC3000)/mock inoculated samples (2h) | 1.119 |
| csn5 (csn5a-2 csn5b)/col | 1.118 |
| TIBA/mock treated seedlings | 1.118 |
| R. solani (AG8)/mock inoculated whole plant samples | 1.116 |
| cold study 6 (Col-0)/20 degrees/18 degrees treated rosette samples (6h) | 1.116 |
| cloransulam-methyl (24h)/mock-treated leaf samples | 1.116 |
| low nitrogen/high itrogen treated rosette samples | 1.115 |
| R. solani (AG2-1)/mock inoculated whole plant samples | 1.115 |
| csn3-1/col | 1.115 |
| Signature profiling tool in the Genevestigator software was used. |  |
