# Supplementary material for: Acetylation of cell wall is required for structural integrity of the leaf surface and exerts a global impact on plant stress responses
Source: Front Plant Sci. 2015 Jul 22;6:550. doi: 10.3389/fpls.2015.00550 (PMC4510344; doi:10.3389/fpls.2015.00550)
Supplement: Supplementary file 5 [file Image1.PDF]

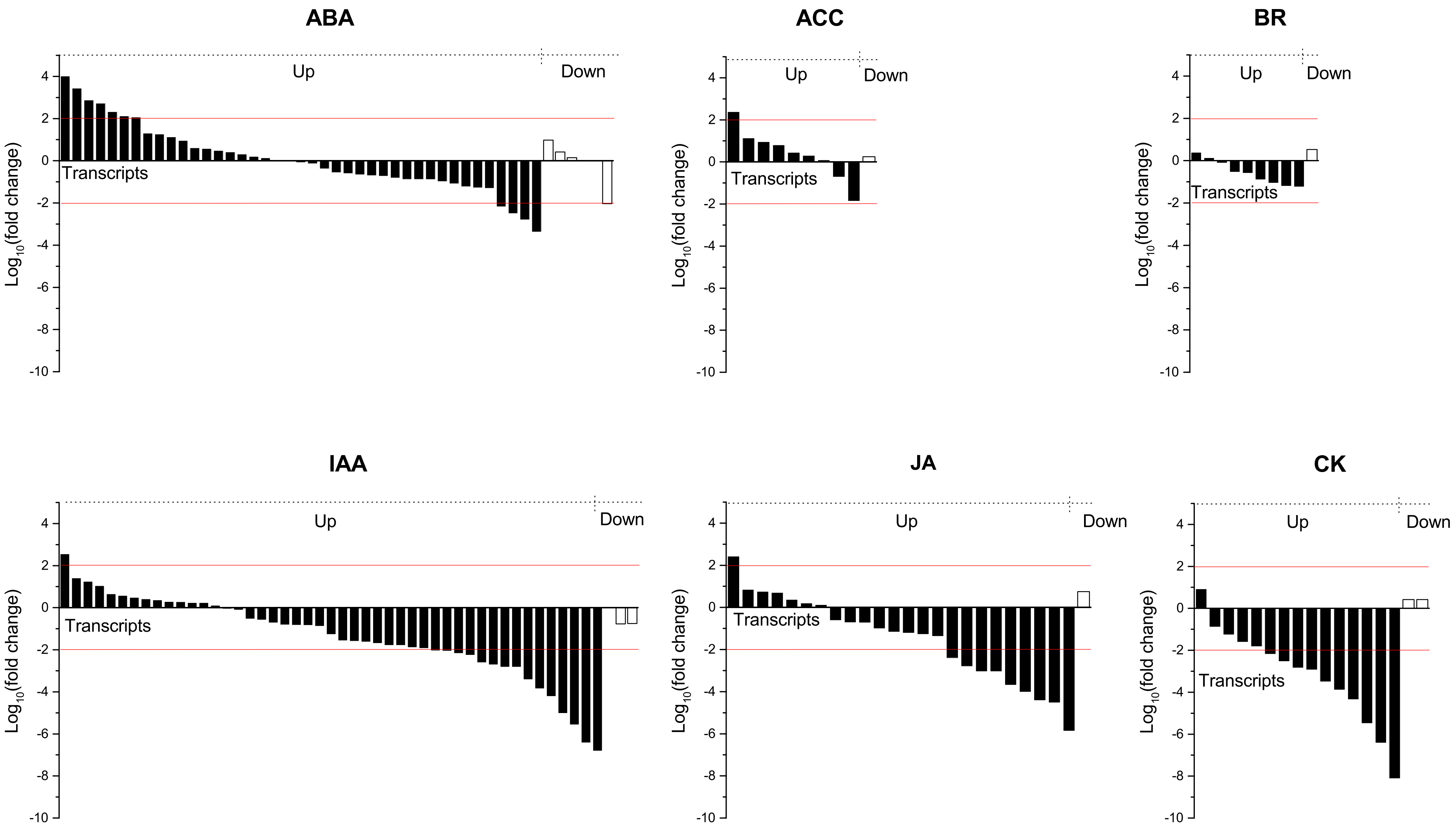

Supplementary Figure 1. Responses of phytohormone-specific transcripts in *rwa2* relative to the wild type. The fold change of transcripts, previously reported to be specifically induced ("Up", shown in black bar) or repressed ("Down", shown in white bar) by a given phytohormone (Nemhauser *et al.* (2006)) are shown. $Log_{10}(2)$ and $Log_{10}(-2)$ are indicated in red lines.
