# Supplementary material for: Acetylation of cell wall is required for structural integrity of the leaf surface and exerts a global impact on plant stress responses
Source: Front Plant Sci. 2015 Jul 22;6:550. doi: 10.3389/fpls.2015.00550 (PMC4510344; doi:10.3389/fpls.2015.00550)
Supplement: Supplementary file 6 [file Image2.PDF]

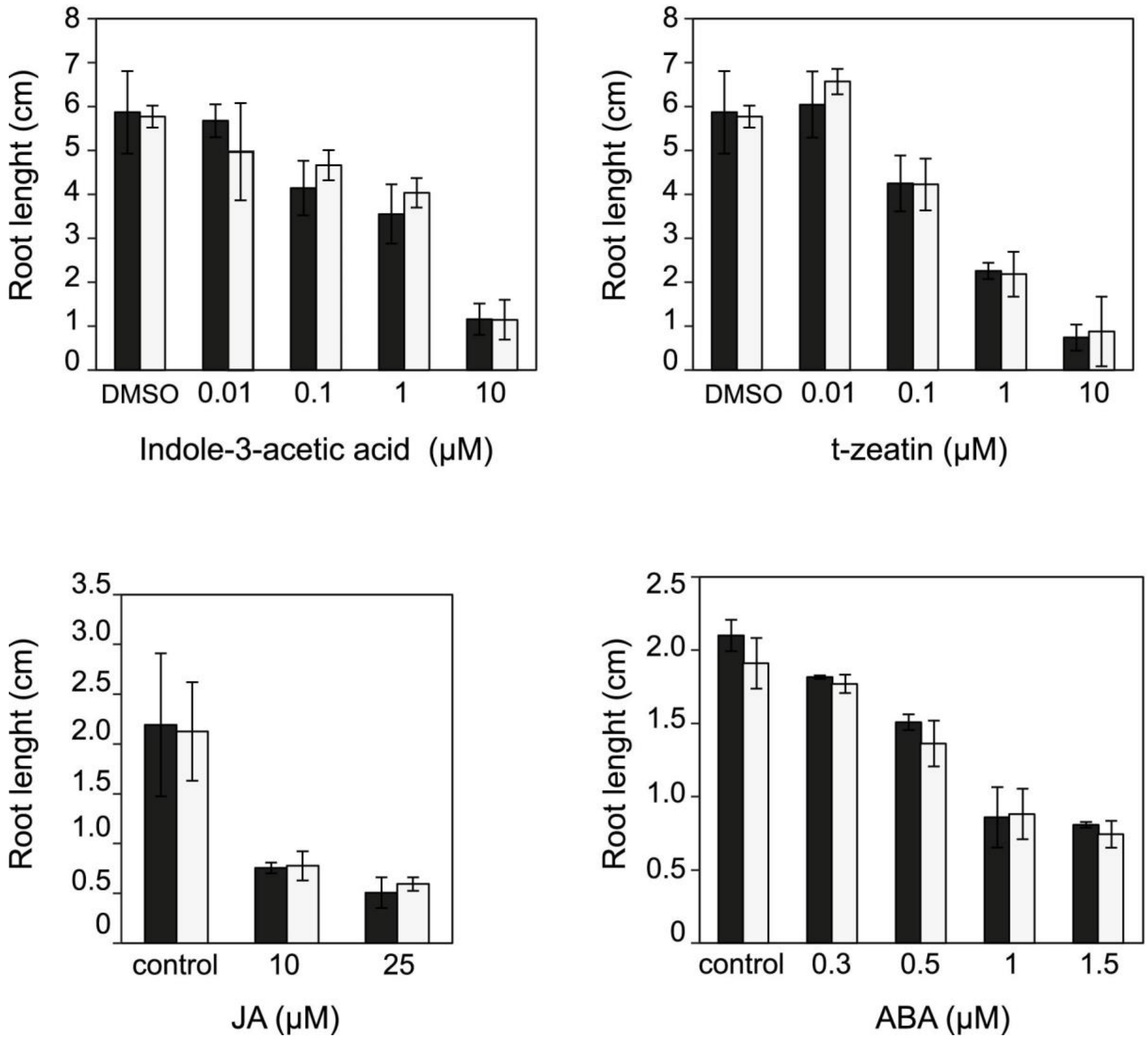

Supplementary Figure 2. *rwa2* seedlings do not have altered response to root growth inhibition by plant hormones.

Root lengths of seedlings grown on vertical agar plates containing JA, indole-3-acetic acid, ABA, and trans-zeatin (CK).
